# Improving Aging in Place for Older Adults with Low Incomes: Perspectives of Home Health Aides

**DOI:** 10.1093/geroni/igaa057.3230

**Published:** 2020-12-16

**Authors:** Momana Jahan, Claudia Hentschel, Sonia Pandit, Mona Akhiary, Tiffany Wisdom-Goulbourne, Barbara Resnick, Carolina Reid, Rebecca Brown

**Affiliations:** 1 University of Pennsylvania, Philadelphia, Pennsylvania, United States; 2 University of Maryland, Baltimore, Maryland, United States; 3 University of California, Berkeley, Berkeley, California, United States

## Abstract

Older adults with low incomes experience disproportionate rates of cognitive and functional impairment and an elevated risk for nursing home admission. Home health aides (HHAs) can help older adults to age in place by optimizing function and engaging them in routine physical activity. Despite this potential role, little is known about HHAs’ perspectives on how to facilitate aging in place for this population. We conducted 6 focus groups with 21 English-speaking and 10 Spanish-speaking HHAs working in Philadelphia and New Jersey. Transcripts were analyzed using qualitative thematic analysis. HHAs described wearing multiple hats and pushing the boundaries of their role as a HHA to provide a “comfortable and safe” environment through nursing and emotional support. Many HHAs shared that they serve as surrogate family, often spending more time in clients’ homes than family members or other healthcare providers. This unique position provides HHAs with valuable insights into clients’ changing health which allows them to detect early warning signs of clients’ functional and cognitive decline, including falls, depression, and confusion. HHAs noted several factors that worsened clients’ decline including a lack of adaptive equipment, social isolation, and limited HHA input into clients’ care plans. They also pointed to factors that facilitated clients’ aging in place, including utilization of community-based services, family support, and communication between healthcare team members. Our findings suggest that HHAs have important insights into improving aging in place for older adults with low incomes and should be incorporated into care planning and intervention delivery.

